# Redefining monetary policy rules: A threshold approach

**DOI:** 10.1371/journal.pone.0252316

**Published:** 2021-05-28

**Authors:** Carmen Diaz-Roldan, María A. Prats, Maria del Carmen Ramos-Herrera

**Affiliations:** 1 Universidad de Castilla-La Mancha, Ciudad Real, Spain; 2 Universidad de Murcia, Murcia, Spain; 3 Universidad Autónoma de Madrid, Madrid, Spain; Universitat Jaume I, SPAIN

## Abstract

In this paper, we try to analyse the extent to which a redefinition of the monetary policy rule would help to avoid the zero-lower bound, as well as to explore the conditions needed to avoid that constraint. To that aim, we estimate the threshold values of the key variables of the policy rule: the inflation gap and the output gap. The threshold model allows us to know which are the turning points from which the relationship between the key variables and the interest rate revert. In the Eurozone countries, we have found that the inflation gap always contributes to increasing the nominal interest rate. On the contrary, the output gap works differently when it reaches values above or below the threshold value, which would favour the reduction of the interest rates towards the zero level.

## 1. Introduction

After the 2007–2008 crisis, there has been *“a shift of policy away from rules as rates were held too low for too long”* [1]. And, after following unconventional monetary policies, nowadays there is a return to the rules-based approach. Some suggestions are being made on reforming inflation targeting to try to reduce the need for unconventional policy instruments, as they are a poor substitute for conventional interest-rate policy in stabilizing the economy [2]. And among the most important issues to be addressed, in the post-crisis time, one concern is the evidence that monetary policy has been constrained by a lower bound on the nominal interest rate [2], as well as by the interaction between monetary and fiscal policies with the objective of stabilization in the short term [3].

The way in which monetary and fiscal policies would interact constitutes a key factor to guarantee the effectiveness of both policies, as addressed by [4] among others. From time to time, the discussions on the interactions between monetary and fiscal policy recover interest by academics as well as policymakers. Mainly at times when monetary (or fiscal) policy seems to be ineffective. Or not as effective as planned. Since the 1990s, the literature on these issues has paid attention to the dominance of one policy, monetary or fiscal, when the policymakers jointly adjust prices and the government budget. Based on the assumption of fiscal dominance, the Fiscal Theory of the Price Level (FTPL) emerged, which stated that endogenous price level adjustment is required to achieve fiscal solvency, even when the central bank has guaranteed legal independence. This policy regime is the so-called “fiscal dominant” in contrast to the traditional or “monetary dominant”. The pioneering contributions on the FTPL were those of [5–12]. In the last decade, after the financial crisis, and, more recently, in the context of the Post-COVID era, authors like [13–16], among others, argue about the irrelevance of the *zero lower bound* (ZLB) constraint assuming a fiscal dominant environment where the central bank is forced to support fiscal solvency. In fact, those contributions are a way of recognizing that when reaching interest values close to zero, an expansive fiscal policy would remedy the ineffectiveness of monetary policy which, at the same time, would help finance fiscal solvency. In other words, a proper interaction of monetary and fiscal policy would mitigate the undesirable effects of low interest rates. Nevertheless, as [15] address, the impact of the ZLB on macroeconomic performance will depend on the macroeconomic context.

The criticisms discussed above, question the effectiveness of traditional Taylor-type rules in dealing with current episodes of low interest rates, leaving unconventional tools behind. But the debate about the new rules and their new targeting proposals have been widely discussed recently in USA, trying to evaluate different alternatives on monetary policy strategies: price level targeting, nominal GDP targeting and different inflation targets [17, 18]. They are all concerned about the ZLB on interest rates and point to the possibility of different targets, other than 2%, for the inflation rate. According to [19], these solutions must be consistent with a Taylor-rule approach.

A concise summary of studies dealing with the costs of effective lower bound (ELB) close to zero, can be found in [20], which addresses the controversy over the cost of raising the inflation target, as a way to avoid the ZLB. Authors like [21] advocate for an inflation target of four percent, while [19] persists in maintaining a target of two percent, even in the presence of low interest rates. Another proposal to deal with low rates, closer to zero, continues to be the use of unconventional monetary policies [22], because the specific rules of monetary policy, used before the financial crisis, do not work in a new era of low interest rates.

In an era of low inflation, [4] proposed to fix an ELB to avoid the ZLB. In that case, the central bank would maintain the official rate at low levels, following periods when the bound were binding. But after the crisis, the evidence is favourable to policy-rules instead of discretion. In the post-crisis world, the expectation of a new implementation of policy rules could be profitable for the economy. The new application of a simple rule, as the Taylor rule or another functional form, would aid as a benchmark for policymakers and not like a mechanical formula for setting the interest rate. [23] purposed an alternative to rules-based policy. This new way could be to use a rule for policy instruments that is equal to a new inflation forecast target.

In this strategy, the central bank would select its official short-term interest rate by a linear combination of its forecasts of different variables; [24] argued the possibility of a time-varying interest rate into a policy rule; [25] pointed out that another way to deal with the zero bound was to go back to money growth rules; but [19] was concerned about the importance of the monetary authorities clarifying their strategy to solve two problems: first, the “obsession” for fixing an inflation target, which could, dangerously, accelerate the economy; and, as a consequence and second, the low attention to other parameters, with the exception of the inflation target, which could diminish the importance of the size of the monetary policy response.

In this paper, we will follow the spirit of [19] who discussed the newer proposals on inflation targeting in the light of concerns about the ZLB, or the ELB, close to zero. Despite the controversy about the costs of modifying the inflation target, or considering other alternatives for conducting monetary policy, concerns about the ZLB remain in force, as can be seen in the last Monetary Policy Report of the Board of Governors of the Federal Reserve System (FED) [26]. According to the FED’s forecasts, the federal funds rates will range from 0 to 0.25 percent until 2024, what could compromise the achievement of other economic policy objectives. In this line, [27, 28] based on the revised statement on longer-run goals and monetary policy strategy of the Federal Open Market Committee (FOMC), proposed some modified monetary policy rules. Those proposals, as well as those stated by [29, 30], aimed to abandon the ZLB, or ELB, after the Great Recession. The main feature of the new rules is the asymmetry: rules are more flexible in periods near to the ZLB. More precisely, rules are *price-level targeting* oriented while federal funds rates are close to zero until an average inflation threshold is reached, and then return to a flexible *inflation targeting*. Specifically, the condition to return to the *inflation targeting* is that inflation reaches a threshold of 2 per cent [29]. In other words, when the increase in is 2 per cent, the ZLB constraint slows down the effectiveness of monetary policy. Summing up, the ZLB continues to worry the monetary authorities, who pay attention to inflation values around its optimal level.

Having those considerations in mind, in this study, our hypothesis would be that by redefining monetary policy rules, a sound monetary policy stance would be possible. Given the positive relationship between both, the inflation gap and the output gap, and the nominal interest rate, shown by the monetary rules, we will try to explore the proper values of the key variables that are compatible with a non-negative value of the interest rate. In this paper, we are concerned with the inflation gap and the output gap values, but not with their weights. Finding the proper value of the parameters involved in the rule is not included among the objectives of this study. Our main concern is related to the values of the variables. And in accordance with the statement of [29], on keeping the ELB until an inflation threshold is reached, we are interested in estimating the threshold values of the variables of the traditional Taylor-type rule. These values, also called trigger points, would inform us which are the turning points from which the relationship between the independent variables and the interest rate would reverse. And how far is the trigger point for the 2 per cent inflation addressed by the FOMC.

To that aim, we will estimate the threshold values of the key variables of the policy rule; the inflation deviations from the inflation target and the output gap, for the 19 countries of the euro area. Our main interest is to explore the necessary conditions to avoid the ZLB, in other words, we will try to find the compatible values with a non-negative value of real interest rates. If some conditions were founded to avoid the ZLB, we could suggest some way to redefine the usual monetary rules in an attempt to guarantee a stable path of interest rates.

Our starting point will be the basic specification of the original Taylor rule [31], which is briefly reviewed in Section 2. Next, we will show the methodological strategy in Section 3. In Section 4 we will show the main results obtained and finally, in Section 5, we present the main conclusions of our paper.

## 2. The Taylor rule: A short reminder

Taylor-type rules, in general, show that short-term deviations of the instrument (x–x*) are a response to deviations from the monetary policy objectives:

$$\left(x–x^{*})=q(z–z^{*}\right)$$ (1)

Deviations of the instrument are a linear combination of the monetary objectives: the stabilization of the level of activity and price stability.

$$\left((i_{t}-\pi_{t})-r^{*})=(\beta -1)(\pi_{t}-\pi^{*})+\gamma (y_{t}{}^{gap}\right)$$ (2)

where *i*_*t*_ denotes the short-term interest rate (official interest rate), r* the equilibrium real interest rate, *π*_*t*_ denotes the inflation rate in period t, π* is the desired long run, or “target,” inflation rate, and $y_{t}^{gap}$ denotes the output gap (the percent deviation of real GDP from its potential level). In other words, deviations of the instrument (real interest rate) from the objective, responds proportionally to the inflation deviations, and the output deviations; or in a different way: the instrument deviations are a linear combination of goals deviations.

Thus, the management of monetary policy is characterized by assigning the targets of output stabilization and price level stabilization (inflation targeting). [31] set the equilibrium interest rate r* equal to 2 and the target inflation rate π* equal to 2, r* = π* = 2, and (*β*−1) = *γ* = 0.5. That is, in principle, the monetary authority would be giving equal importance to price stability as to the macroeconomic stabilization.

Rearranging terms, Taylor’s rule says that the short-term interest rate should equal one-and-a-half times the inflation rate plus half the output gap plus one.

$$i_{t}=1+1.5\pi_{t}+0.5{y_{t}}^{gap}$$ (3)

Or in general terms,

$$i_{t}=\alpha +\beta \pi_{t}+\gamma {y_{t}}^{gap}$$ (4)

where *α* would proxy, together with other exogenous variables, the nominal interest rate goal.

The value of the coefficients represents the weight that the central bank gives to the inflation rate and the level of activity in its objective function. If the parameters were different, the rule would indicate which objective (inflation or economic growth) has more weight in determining monetary policy. Thus, with *β*>1 the central bank has to adopt an anti-inflationist monetary policy by raising the real interest rate in order to slow down the economy and reduce inflationary pressures contributing to macroeconomic stabilization [32]. For the output gap coefficient *γ*>0, the central bank has to adopt a countercyclical monetary policy, increasing the official interest rate by a particular amount when real GDP exceeds potential GDP and decreasing the interest rate therein amount when real GDP falls below potential GDP [33].

When setting the nominal interest rate, the central bank uses a rule similar to:

$$i_{t}=\alpha +\beta \pi_{t}^{e}+\gamma {y_{t}}^{gap}$$ (5)

That is, the central bank decides the nominal interest rate for the period t based on the target nominal interest rate, modified according to the deviation of inflation expectations and the deviation of output expectations from its long-term tendency. If the central bank gives priority to the inflation target, and does not include output stability objective of monetary policy, then it implies that *γ* = 0, and then the monetary rule would be as follows:

$$i_{t}=\alpha +\beta \pi_{t}^{e}$$ (6)

That is similar to the monetary policy rule followed by the European Central Bank. Assuming rational expectations, the expected inflation rate would match the true inflation rate, *π*_*t*_, except for a random prediction error, *ε*_*t*_,

$$\pi_{t}^{e}=\pi_{t}+\varepsilon_{t}$$ (7)

So, we would get

$$i_{t}=\alpha +\beta \pi_{t}+\omega_{t}$$ (8)

where $\omega_{t}=\beta \pi_{t}^{e}$

In the rest of the paper, rearranging Eq (2), we will consider the Taylor-type rule given by the following equation:

$$i_{t}=\alpha +(\beta -1)(\pi_{t}-\pi^{*})+\gamma ({y_{t}}^{gap})$$ (9)

Recall that *α* would proxy, together with other exogenous variables, the nominal interest rate goal. And where we assume that (*β*−1)>0, and *γ*>0, what assures macroeconomic stabilization.

## 3. Econometric methodology

In our attempt to check the robustness of the relationships involved in the usual monetary rules, our starting point will be the traditional Taylor-type rule [31] which is equivalent to the Eq (9) above. To estimate the threshold values of the variables in the equation, we will use the data, provided by Eurostat, on inflation, output-gap, and interest rates for the 19 countries of the euro area (Austria, Belgium, Cyprus, Estonia, Finland, France, Germany, Greece, Ireland, Italy, Latvia, Lithuania, Luxembourg, Malta, the Netherlands, Portugal, Slovakia, Slovenia, and Spain) from 1999: Q1 to 2019: Q3.

In S1 Fig we can observe the trajectory of each variable in each country, as well as the average values of these variables in the European Monetary Union (EMU) as a whole. As can be seen the trajectory of the ten main EMU countries (Austria, Belgium, Finland, France, Germany, Italy, Luxembourg, the Netherlands, Portugal, and Spain) is quite similar, in terms of the reference variables, unlike the rest of the countries; which determines that the average data for the EMU-19, which after all is what guides the ECB’s monetary policy, hides a notable heterogeneity.

Our estimation method will be panel data. According to [34], the main advantages of using panel data techniques are the following: greater capacity to capture complex relationships; better inference of the model parameters; higher ability to control for individual unobserved heterogeneity; simpler computation and statistical inference, and more informative results. See for instance [35] to deeply analyse the strengths and weaknesses of using panel data.

A necessary requirement is to check the stationarity of our variables due to the fact that, if they do not fulfil this condition, the asymptotic analysis will not be valid, and it would not be possible to carry out hypothesis tests on parameters. We apply several unit roots tests on panel datasets in order to achieve robust results. We implement [36, 37], Fisher-type [38–40] tests. In all cases the null hypothesis is that the specific variable is non-stationary (it is integrated of order 1, I (1)).

The results are shown in Table 1. As can be seen, we decisively reject the corresponding null hypothesis that indicates that the short-term interest rate, the inflation gap and the output gap are stationary in levels, that is, they are integrated of order zero, I (0). In other words, it is not necessary to differentiate the variables to achieve the essential properties to apply this methodology. Our results are very robust because, except for the [37] test in levels, the interest rate is stationarity in all cases. The same happens for the [39] test with trend to the inflation gap.

**Table 1:** Results of unit roots tests.

Test Statistic	Interest rate	Inflation gap	Output gap
**LLC**			
*Level*	-15.1875	-2.6393	-10.8479
*p-value*	(0.0000)	(0.0042)	(0.0000)
*Trend*	-9.2947	-0.2516	-6.4144
*p-value*	(0.0000)	(0.4007)	(0.0000)
**HT**			
*Level*	0.8424	0.9088	-0.0211
*p-value*	(0.0000)	(0.0000)	(0.0000)
*Trend*	0.8157	0.8883	-0.0324
*p-value*	(0.0000)	(0.0401)	(0.0000)
**Br**			
*Level*	2.6440	-6.2085	-13.6634
*p-value*	(0.9959)	(0.0000)	(0.0000)
*Trend*	-1.5148	-4.4185	-16.6233
*p-value*	(0.0649)	(0.0000)	(0.0000)
**IPS**			
*Level*	-11.8350	-3.7310	-25.5719
*p-value*	(0.0000)	(0.0001)	(0.0000)
*Trend*	-10.3249	-4.7836	-25.8422
*p-value*	(0.0000)	(0.0000)	(0.0000)
**Fisher**			
*Level*	31.9164	15.2551	45.0847
*p-value*	(0.0000)	(0.0000)	(0.0000)
*Trend*	28.0992	13.9102	37.5827
*p-value*	(0.0000)	(0.0000)	(0.0000)

Note**: HT** holds for [15], **Br** for [16], **Fisher** for Fisher-type [17], **LLC** for [18] and **IPS** for [19] test, respectively.

After checking the stationarity of the variables, we analyse whether the interest rate behaves differently above or below a certain threshold. In other words, we study at what point the relationship between our explanatory variables and our dependent variable should be different. For this reason, our purpose is to implement a dynamic panel threshold model to estimate the potential asymmetric impact of the exogeneous variables: the inflation gap and the output gap.

The single-threshold model can be expressed in the following form:

$$y_{it}=\alpha_{i}+\delta_{1}X_{it}(q_{it}<\tau )+\delta_{2}X_{it}(q_{it}\geq \tau )+e_{it}$$ (10)

where *α*_*i*_ are the country-specific intercepts, *q*_*it*_ is the threshold variable and *τ* is the threshold parameter or trigger point.

An alternative for the above equation is:

$$y_{it}=\alpha_{i}+X_{it}(q_{it},\tau )\delta +e_{it}$$

where $X_{it}(q_{it},\tau )=\left\{ \begin{array}{c} X_{it}I(q_{it}<\tau )\\ X_{it}I(q_{it}\geq \tau ) \end{array}\right.$ and I(.) is an indicator function.

According to the literature, the *τ*′*s* estimator is achieved when the residual sum of squares (RSS) is minimized as follows, based on a subset of the threshold variable (*q*_*it*_):

$$\hat{\tau }=\arg\;\min\;S_{1}(\tau )$$

Then, the ordinary least squares estimator of *δ* is:

$$\hat{\delta }={\left\{X^{*}{\left(\tau \right)}^{\prime }X^{*}(\tau )\right)}^{-1}\{X^{*}{\left(\tau \right)}^{\prime }y^{*}\}$$

where y* and X* are within-group deviations.

In particular, this procedure focuses on several quantiles of the threshold variable, that is, in a specific interval ($\underline{\tau },\bar{\tau }$). It is well known the nuisance parameter problem associated when the threshold parameter is unknown due to the fact that estimation and inference are more complex because the distribution of the *τ* estimator is nonstandard. [41] solves this concern by showing that $\hat{\tau }$ is a consistent estimator for *τ*.

To determine if there is a threshold effect, it is necessary to apply a simple test to help us to clarify whether the coefficients are different in each regime. For this reason, we implement the following test in which the linear model is captured in the null hypothesis and the single-threshold model is collected in the alternative hypothesis as can be seen:

$$H_{0}:\delta_{1}=\delta_{2}\;H_{1}:\delta_{1}\neq \delta_{2}$$

and the corresponding F statistic is computed as:

$$F_{1}=\frac{\left(S_{0}-S_{1}\right)}{{\hat{\sigma }}^{2}}$$

in which *S*_0_ is the RSS of the linear model. It is important to note that the critical values of the F statistic are based on the bootstrap methodology to analyse the significance of the threshold.

Additionally, in this study we check the possibility of having multiple-threshold models. To carry out this procedure, we do it sequentially, that is, we implement another test in which the null hypothesis is linked with a single-threshold model and the alternative hypothesis is the double-threshold model which can be expressed as follows:

$$y_{it}=\alpha_{i}+X_{it}(q_{it}<\tau_{1})\delta_{1}+X_{it}(\tau_{1}\leq q_{it}<\tau_{2})\delta_{2}+X_{it}(q_{it}\geq \tau_{2})\delta_{3}+e_{it}$$ (11)

where *τ*_1_ *and τ*_2_ are the two threshold values and *δ*_1_, *δ*_2_ *and δ*_3_ capture the three impacts for each regime.

The first step is to estimate the threshold estimator *τ*_1_ linked to the single-threshold model and compute the associated RSS ($S_{1}({\hat{\gamma }}_{1})$). The second step is related to work out the second threshold and its confidence interval:

$${\hat{\tau }}_{2}^{r}=\arg\;\min\left\{S_{2}^{r}(\tau_{2})\right\}$$

$$S_{2}^{r}=S\{min({\hat{\tau }}_{1},\tau_{2})max({\hat{\tau }}_{1},\tau_{2})\}$$

$${LR}_{2}^{R}(\tau_{2})=\frac{\left\{S_{2}^{r}(\tau_{2})-S_{2}^{r}({\hat{\tau }}_{2}^{r})\right\}}{{\hat{\sigma }}_{22}^{2}}$$

Finally, the way to determine the F statistic is as follows:

$$F_{2}=\frac{\left\{S_{1}({\hat{\tau }}_{1})-S_{2}^{r}({\hat{\tau }}_{2})\right\}}{{\hat{\sigma }}_{22}^{2}}$$

## 4. Empirical results and discussion

Table 2 offers the results of the dynamic panel threshold model for the 19 countries of the euro area from 1999: Q1 to 2019: Q3. The upper part of this table shows the estimated output gap and the inflation threshold respectively and the corresponding 95% confidence interval. In particular, the estimated output gap threshold is -2.4289%, which is statistically significant at the 1% level and the 95% confidence interval is [-2.9391, -2.4071].

**Table 2:** Results of output gap and its impact on interest rate.

	Model 1 (Output gap)	Model 2 (Inflation gap)
**Threshold estimates (**$\hat{\tau }$**)**	-2.4289	-2.8667
Significance of threshold p-value	0.0200	0.0962
95% Confidence interval	[-2.9391, -2.4071]	[-3.3333, -2.8000]
**Impact of threshold variable on interest rate:**		
${\hat{\delta }}_{1}$	-0.1394***	0.1744**
	(0.0315)	(0.0924)
${\hat{\delta }}_{2}$	0.1943***	0.6598***
	(0.0363)	(0.0265)
**Impact of control variables on interest rate:**		
Inf_gap	0.5969***	
	(0.0253)	
Output_gap		0.0099
		(0.0230)
Constant	2.2881***	2.2060***
	(0.0585)	(0.0593)
	(0.0038)	
N	1577	1577
*R*^2^ within	0.2968	0.2879
*R*^2^ between	0.6793	0.2703
*R*^2^ overall	0.3382	0.2857

Source: Own elaboration. Notes: In the ordinary brackets below the parameter estimates are the corresponding *z*-statistics, computed using [42] heteroskedasticity-robust standard errors. In the square brackets below the specification tests are the associated *p*-values.

*, ** and *** indicate significance at 10%, 5%, and 1% respectively.

The regime-dependent coefficients of these two explanatory variables on the short-term interest rate are shown in the middle part of Table 2. In Model 1, ${\hat{\delta }}_{1}$, and ${\hat{\delta }}_{2}$ represent the marginal effects of the output gap on the interest rate when the output gap is below and above the estimated threshold value, respectively. Specifically, the output gap is negatively correlated with the interest rate when the output gap is less than the threshold (${\hat{\delta }}_{1}=-0.1394$), however the opposite occurs when it is higher than the threshold (${\hat{\delta }}_{2}=0.1943$). This implies that an increment in the deviation of economic growth from the objective above the estimated threshold may lead to an increase in the interest rate.

The estimated threshold value, or trigger point value, of -2.4289 means that there is a turning point from which the relationship between the output gap and the interest rate behaves differently, showing two different regimes, when the output gap increases above or below 2.4289%. The unknown threshold value is between the values [-2.9391, -2.4071] with 95% confidence. That is, when the output gap is below the threshold there is a negative relationship between both threshold variables (${\hat{\delta }}_{1}=-0.1394$), and when the output gap reaches the threshold, values above already generate a positive relationship (${\hat{\delta }}_{2}=0.1943$).

Additionally, by looking at the absolute value of these coefficients we can interpret that the correlation is stronger above the threshold value. Finally, the coefficients of the control variables are presented in the lower part of this table, which in this case means that the inflation gap is highly significant and positively correlated with the interest rate.

Considering the inflation gap as the threshold variable (Model 2), the empirical results suggest that the estimated threshold is -2.8667% and the confidence interval is [-3.3333, -2.8000]. The same previous procedure is applied to interpret the results with respect to the marginal effects, it means that ${\hat{\delta }}_{1}$, and ${\hat{\delta }}_{2}$ reveal how much the interest rate changes when the inflation gap is below and above the estimated threshold value, respectively. Therefore, the estimated threshold value or trigger point value of -2.8667 means that there is a turning point from which the relationship between the inflation gap and the interest rate behaves differently. And the unknown threshold value is between the values [-3.3333, -2.8000] with 95% confidence. But in this case, there are not two different regimes when the inflation gap increases above 2.8667%, the only difference is that the magnitude of the impact is different.

The coefficients of the effect of the inflation gap on the interest rate are significant and plausibly confirmed. Therefore, there is no change in the relationship between the inflation gap and the interest rate. That is, although the figures of inflation gap are above or below the estimated threshold value of -2.8667, as the inflation gap increases, the interest rate will increase. But when the inflation gap is below the threshold, the marginal effects on the interest rate are given by (${\hat{\delta }}_{1}=0.1744$); on the contrary, the marginal effect are (${\hat{\delta }}_{2}=0.6598$) being stronger above the threshold value.

Our next purpose will be to question whether there is a single trigger point or more than one, checking the possibility of having multiple-threshold models. The results associated with the analysis of multiple-threshold models are presented in Table 3. We compare between the linear, or single-threshold model, and the single-threshold, or double-threshold, model and successively. Specifically, for both variables we reject the null hypothesis of linear model being more appropriate the single-threshold model. When we compare between single and double, we do not have enough statistically evidence to reject the null hypothesis, henceforth we rule out the possibility of double-threshold model. In other words, the results suggest that it is better to consider a single threshold rejecting the possibility of two and therefore more thresholds.

**Table 3:** Multiple-threshold models of each explanatory variable and its impact on interest rate.

**Output gap and its impact on interest rate**							
**Threshold estimator (level = 95):**							
**Model**		**Threshold**		**Lower**		**Upper**	
Th_1		-2.4289		-2.9391		-2.4071	
Th_21		-2.4289		-2.5395		-2.4071	
Th_22		2.6079		0.4122		2.7786	
Threshold-effect test:							
Threshold	RSS	MSE	F-stat	Prob	Crit10	Crit5	Crit1
Single	6143.79	4.1123	43.18	0.0100	26.0691	29.8493	40.7771
Double	6094.08	4.0790	12.19	0.6833	26.8897	33.6583	37.2343
**Inflation gap and its impact on interest rate**							
**Threshold estimator (level = 95):**							
**Model**		**Threshold**		**Lower**		**Upper**	
Th_1		-2.8667		-3.3333		-2.8000	
Th_21		-2.8667		-3.3333		-2.8000	
Th_22		-0.9333		-0.9667		-0.9000	
Threshold-effect test:							
Threshold	RSS	MSE	F-stat	Prob	Crit10	Crit5	Crit1
Single	6227.03	4.1680	22.63	0.0962	23.6587	29.1344	43.2392
Double	6199.00	4.1493	6.76	0.3215	13.1308	17.5517	24.6457

Source: Own elaboration. Notes: In the threshold-effect test we show the RSS, the mean squared error (MSE), the F statistic (F-stat), the probability value of the F statistic (Prob) and the critical values at 10%, 5% and 1% significance levels (Crit10, Crit5 and Crit1, respectively). Th_1 denotes the estimator in single-threshold model. Th_21 and Th_22 refers to the two estimators in the double-threshold model. Single considers that the null hypothesis is the linear model and the alternative hypothesis is the single-threshold model and Double captures a single-threshold model in the null hypothesis and a double-threshold model in the alternative hypothesis.

Summing up, throughout the period 1999 to 2019 in the euro area countries: the inflation gap values have always contributed to the increase in nominal interests; the marginal effects have been much stronger when the inflation gap has increased above 2.8667%; and the output gap values have not always lead to the same effect on interest rates. When the output gap increases below or above 2.4289%, the relationship between the output gap and the interest rate shows two different regimes. When the output gap is below the relationship is negative, and positive for values above, being the marginal effects slightly stronger in this second regime. In any case, we rule out the possibility of a double-threshold model. Table 4 summarizes the related effects on the nominal interest rate.

**Table 4:** Marginal effects of the two regimes defined by the estimated threshold values.

	Marginal effects on the interest rate
**Inflation gap (*π***_***t***_−π*)	
**Regime 1: (*π***_***t***_−π*) > − 2.8667	0.6598
**Regime 2: (*π***_***t***_−π*)<− 2.8667	0.1744
**Output gap** *y*_*t*_^*gap*^	
**Regime 1:** *y*_*t*_^*gap*^> − 2.4289	0.1943
**Regime 2:** *y*_*t*_^*gap*^<− 2.4289	−0.1394

Source: Own elaboration according to the results of Table 2.

Do our results have any implication for redefining monetary rules? According to the design of the Taylor-type monetary rules, the target inflation rate π* is presumably fixed at a value lower than the inflation rate records (see [19] for a brief discussion on the setting of 2% as a target). In this way, it would be guaranteed that the short-term deviations of inflation from the target would be positive. We could say that, in some way, monetary authorities design the *inflation target* as a question of minimizing an inflation gap that is expected to be positive. In this way, changes of the targeted inflation rate value would mean a new simply monetary policy (expansive or contractive) that could adjust the monetary directions to the evolution of the inflation rate records.

In contrast, the stabilization goal of minimizing the output gap works differently. GDP will rise above, or fall below, potential GDP causing opposite effects on the nominal interest rate, according to the Taylor-type monetary rules. Keeping the output gap coefficient *γ*>0 guarantees that the central bank would adopt a countercyclical monetary policy. However, the monetary authorities cannot redefine potential GDP, as they can redefine the inflation rate target.

What do these considerations mean in terms of redefining monetary rules? Do they have any implication when trying to avoid the ZLB? In this paper, the threshold approach has allowed us to define two different regimes for both the inflation gap and the output gap. According to our results, when the output gap increases below 2.4289%, the relationship with the nominal interest rate is negative, leading to a negative marginal effect that implies a reduction of − 0.1394 points in the interest rate. Would this mean a risk of reaching negative interest rates? Not necessarily. In this sense, the weights for the inflation rate differential, (*β*−1), and for the output gap, *γ*, as well as the value of *α*, in terms of Eq (9), play a key role.

## 5. Conclusions

In this paper, following the proposals summarized by [19], as well as those stated by [29], we have tried to study the implications of one of the ideas aimed to redefine the usual monetary rules, to guarantee a sound and stable monetary policy. Given the evidence that monetary policy has been constrained by a lower bound of the nominal interest rate, we have intended to study to what extent the values of the inflation gap and the output gap would contribute to being or not close to the ZLB. In other words, we have explored the proper values of the key variables that are compatible with a non-negative value of the interest rate. In particular, we have explored the turning points from which the relationship between the independent variables and the interest rate would be reversed, and how far is the trigger point for the 2 per cent inflation rate addressed by the FOMC. With this objective in mind, we have estimated the threshold values of the inflation gap and the output gap. The threshold approach has allowed us to know if the interest rate behaves differently when the inflation gap and the output gap turn out to be above or below the estimated threshold value.

Our results show that, in the euro area from 1999 to 2019, the inflation gap values have always contributed to increasing the nominal interest rate. The effects are much stronger when the inflation gap has increased above the estimated threshold value. The turning point for the average inflation of the Eurozone is a 2.8 per cent, higher than the 2 per cent advised by the FOMC of the Federal Reserve.

However, when the output gap values have been below or above the threshold value, we can observe two different regimes that lead to a negative or positive effect on the nominal interest rate. This would imply, that when including the minimization of the output gap as monetary policy target, the central bank should monitor deviations of GDP from potential GDP. The nature of this variable, which captures business cycles, leads to opposite effects on interest rates depending on the economic growth trends. And potential GDP cannot be redefined as the inflation target could be. So, it would be advisable to pay attention to the evolution of the output.

As a natural extension of our findings, given the threshold values of the key variables, we will try to analyse what would be the adequate parameters of the monetary rules compatible with a non-negative nominal interest rate.

## Supporting information

S1 Fig Interest rate, output gap and inflation gap in EMU (%). (TIF)

S1 File (XLSX)
