# Supplementary figures and images for: Redefining monetary policy rules: A threshold approach
Source: PLoS One. 2021 May 28;16(5):e0252316. doi: 10.1371/journal.pone.0252316 (PMC8162680; doi:10.1371/journal.pone.0252316)

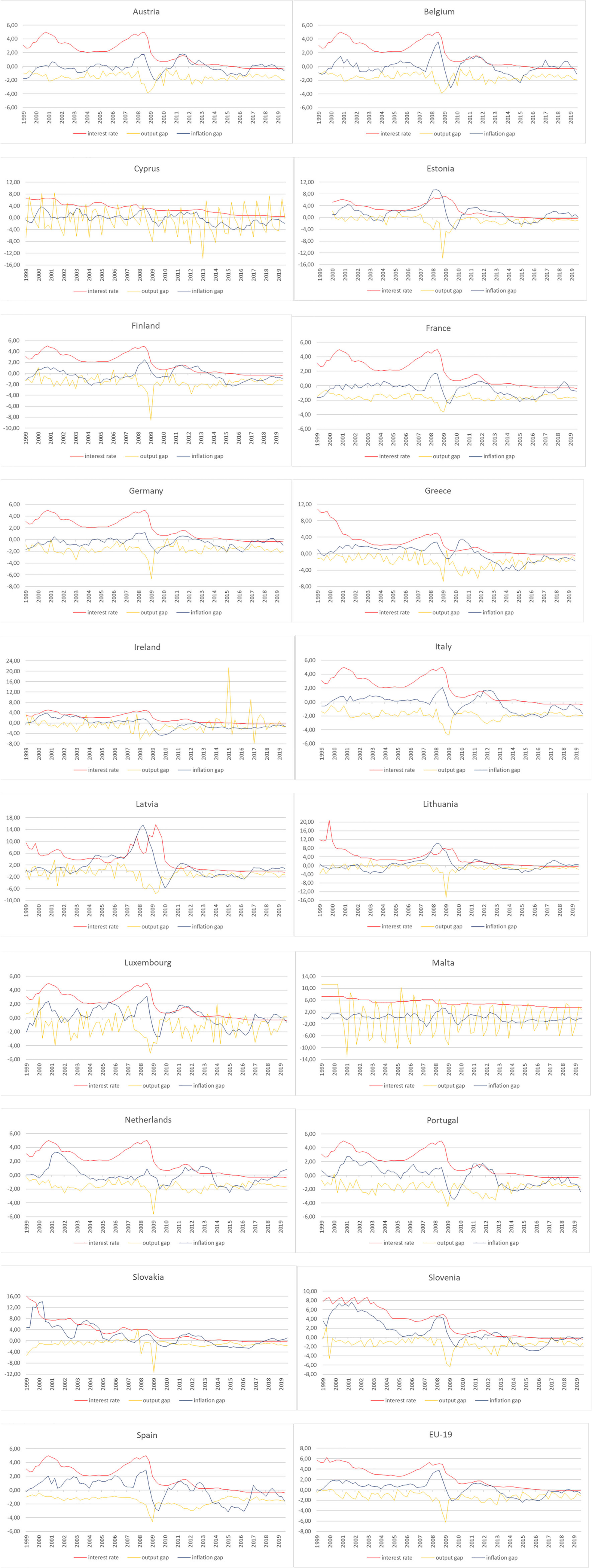

Supplement: S1 Fig — (TIF) [file pone.0252316.s001.tif]
